# *Vagococcus fluvialis* isolation from the urine of a bladder cancer patient: a case report

**DOI:** 10.1186/s12879-024-09082-w

**Published:** 2024-02-26

**Authors:** Qian Chen, Siwen Tan, Sheng Long, Kaixuan Wang, Qi Liu

**Affiliations:** 1 Department of Surgery, Hunan Provincial People’s Hospital, The First Affiliated Hospital of Hunan Normal University, 410005 Changsha, Hunan P.R. China; 2 Department of Comprehensive Surgery, Hunan Provincial People’s Hospital, The First Affiliated Hospital of Hunan Normal University, 410005 Changsha, Hunan P.R. China; 3 Department of Clinical Laboratory, Hunan Province People’s Hospital, The First Affiliated Hospital of Hunan Normal University, 410005 Changsha, Hunan P.R. China

**Keywords:** *Vagococcus fluvialis*, Urine, MALDI-TOF MS, Bacterial identification, Rare

## Abstract

*Vagococcus fluvialis* infection is rare in humans, and there is limited research on the clinical manifestations and antimicrobial susceptibility testing of *Vagococcus fluvialis* infection. Here, We isolated *Vagococcus fluvialis* from the urine samples of bladder cancer patients at Hunan Provincial People’s Hospital, and it is the first reported case of *Vagococcus fluvialis* isolated from the urine. The fully automated microbial identification system and matrix-assisted laser desorption/ionization time-of-flight mass spectrometry (MALDI-TOF MS) identified the bacterium as Vagococcus fluvialis with a confidence level of 99.9%. The VITEK-2Compact fully automated microbial susceptibility analysis system indicated that it was most sensitive to tigecycline, vancomycin, quinupristin/dalfopristin, linezolid, and showed moderate sensitivity to erythromycin, levofloxacin, ciprofloxacin, ampicillin/sulbactam, and tetracycline. Additionally, it exhibited synergy when combined with high-level gentamicin and vancomycin, showing sensitivity. However, it displayed poor activity against penicillin and furanth. According to our knowledge, this is the first study to isolate and identify *Vagococcus fluvialis* from the urine of bladder cancer patients and the systematically reviewed other reported *Vagococcus* infections on human, which provide an experimental basis for guiding the rational use of drugs in the clinical treatment and diagnose of *Vagococcus fluvialis* infection and related pathogenic mechanism research. Meanwhile, we have systematically reviewed other reported.

## Introduction

*Vagococcus fluvialis* is a species in the genus *Vagococcus*, which is a particular type of Gram-stain positive bacterium that is spherical and similar to *lactococci*. It can react with anti-sera against *lactococci* [1]. It was first reported by Collins in 1989 [2]. To date, this bacterium has been isolated from human clinical specimens in several locations [3–15], including blood, ascites, gallbladder fluid, wound puncture fluid, and root canals. Reports on the pathogenicity of *Vagococcus fluvialis* are mostly derived from marine animals such as otters, seals, bass, salmon [16–19], and rainbow trout, as well as infections in domestic animals such as pigs and cows [20]. This indicates that *Vagococcus fluvialis* can infect a variety of animals and can also serve as a source of infection in humans. *Vagococcus fluvialis* isolated from urine samples is rarer, and there are no relevant reported cases in China and abroad. There are different symptoms and physical findings depending on the different areas of the body that are affected.

*Vagococcus fluvialis* infection is not common in humans, and there is limited research on the clinical manifestations and antimicrobial susceptibility testing of *Vagococcus fluvialis* infection. Here, We isolated *Vagococcus fluvialis* isolated from the urine samples of bladder cancer patients at Hunan Provincial People’s Hospital. Through initial verification using colony morphology observation on blood agar plates, Gram staining microscopic analysis, and biochemical experiments with an automatic microbiological detection instrument, this pathogenic bacterium is likely to belong to the rare genus of *Vagococcus fluvialis*. To ensure accurate identification, in conjunction with relevant literature reports, Matrix-Assisted Laser Desorption/Ionization Time-of-Flight Mass Spectrometry (MALDI-TOF MS) was employed, proving advantageous in identifying rare bacterial species. This study further confirmed the identification of the pathogenic bacterium as riverine roaming cocci through MALDI-TOF MS technology. Meanwhile, we have systematically reviewed other reported *Vagococcus* infections. It lays an experimental foundation for guiding clinically rational drug use for infections caused by *Vagococcus fluvialis* and exploring related pathogenic mechanisms.

## Materials and methods

### Patients and samples

A 60-year-old man presented at Hunan Provincial People’s Hospital with a small amount of blood in his urine, accompanied by urinary frequency, urgency, and pain for over a year. He also had a low-grade fever and blood in his urine constantly for the past 4 days. He did not experience chills, chest tightness, shortness of breath, or difficulty breathing. His mental state, diet, sleep, and bowel movements were normal. Laboratory tests showed that he had a total of 1573.7 red blood cells per microliter, 199.2 white blood cells per microliter, 655.2 bacteria per microliter, a 3 + occult blood in the urine, a 1 + urinary leukocyte esterase, and a 1 + urinary protein. The results suggest that the patient may have a urinary tract infection. Abdominal color Doppler ultrasound showed a solid mass lesion in the anterior and left walls of the bladder. A CTU of the urinary system suggested a bladder mass, with a possibility of malignant tumor originating from the urachus, involving the serosal layer.

### Urinary pathogen cultivation and identification

#### Isolation and cultivation

Urine specimens were collected and inoculated onto Columbia blood agar plates and MacConkey agar plates with alpha-lytic protease inhibitors. The plates were then incubated at 35 °C with 5% CO2 in a incubator-CO2 for 24–48 h, and the colony morphology was observed.

### Microscopic examination

Picking a single colony from a blood agar plate of Colombia, preparation of a slide, performing Gram-stain, and microscopic examination using an oil immersion lens.

### Strain identification

Picking a pure culture of the test bacterium from a blood agar plate of Colombia, preparing a bacterial suspension of 0.5 McFarland units, based on Gram-stain results, selecting the Gram-stain positive bacteria identification card (GP card) and the Gram-stain positive bacteria susceptibility card (GP67 card) from the VITEK-2 Compact fully automated microbial identification and susceptibility analysis system for testing. The test results are determined using the AES advanced analysis system. Additionally, the test bacterium is streaked onto the target plate of the VITEK MS fully automated rapid microbial mass spectrometry detection system, and 1.0µL CHCA matrix solution is added. After the matrix solution is dried, the sample is analyzed using the machine. Peak comparison and integration calculations are performed using the VITEK MSIVD library, and the identification results are analyzed using the advanced spectrum classifier (ASC) method for high-level mass spectrometry analysis.

### Drug sensitivity experiment

Select pure cultures of the test bacteria from Colombia blood agar plates. Prepare a bacterial suspension with a concentration of 0.5 McFarland units. Based on the Gram-stain results, choose the Gram-stain positive bacterial drug sensitivity card, GP67 card, and load it into the VITEK-2 Compact fully automated microbial identification and drug sensitivity analysis system for testing.

## Results

### Cultivation results

**Fig. 1:**
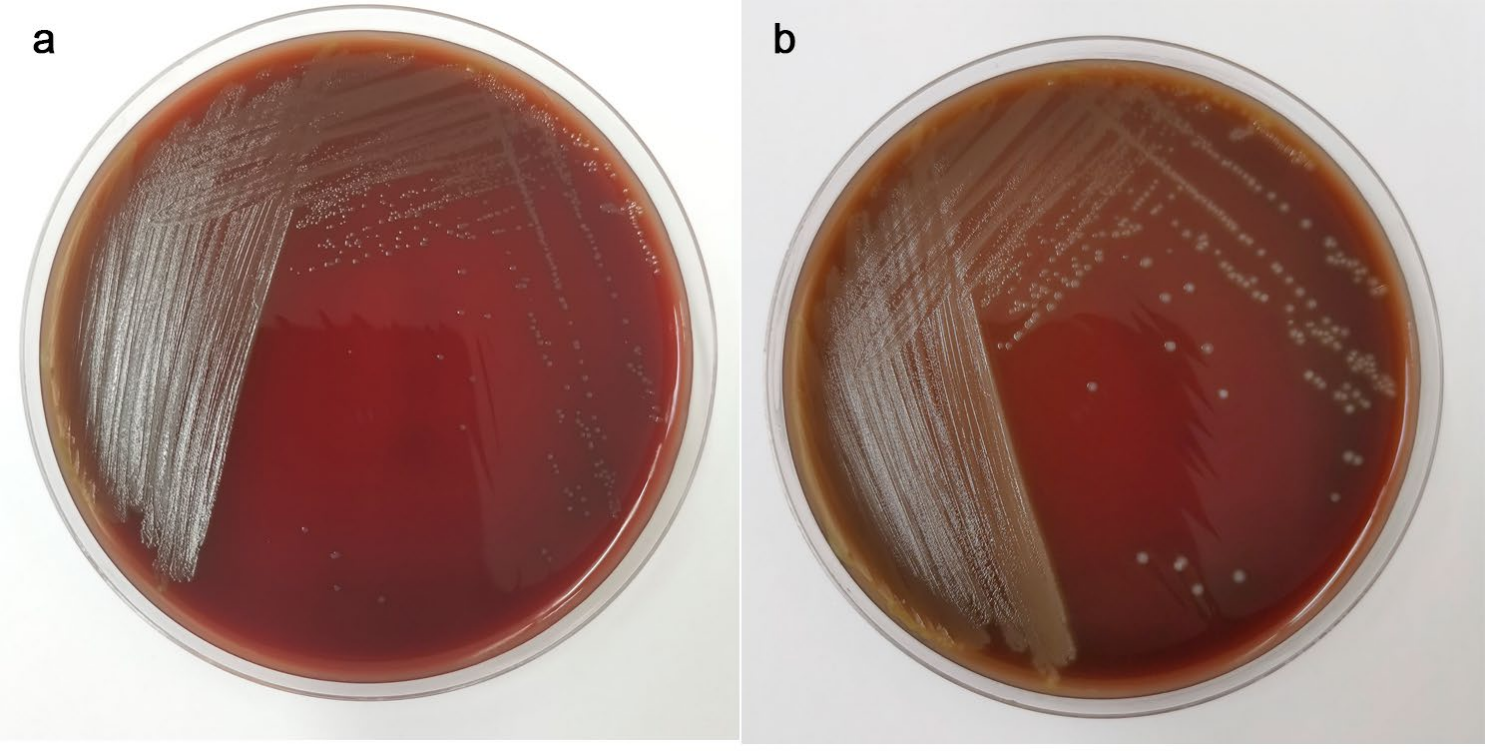
Colony morphology on blood agar plate *Note*: (left) the colony morphology of the test bacteria after 24 h of incubation on blood agar plates; (right) the colony morphology of the test bacteria after 48 h of incubation on blood agar plates

After 24 h of cultivation, the tested bacteria grew into small, pointed, grayish-white, smooth, and raised colonies. There was slight α-hemolysis. After pure cultivation, the colony morphology was more pronounced after 48 h, showing α-hemolysis. The hemolytic zone gradually increased, as shown in Fig. 1.

### Gram-stain microscopic examination results

**Fig. 2:**
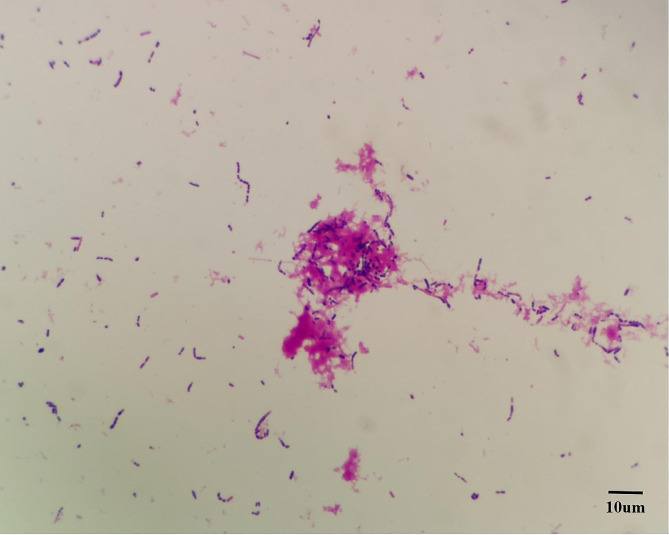
The morphology under Gram-stain (×1000)

Gram-stain showed positive *cocci*, relatively small in size, arranged in pairs or chains, as shown in Fig. 2.

### Mass spectrometry identification

**Fig. 3:**
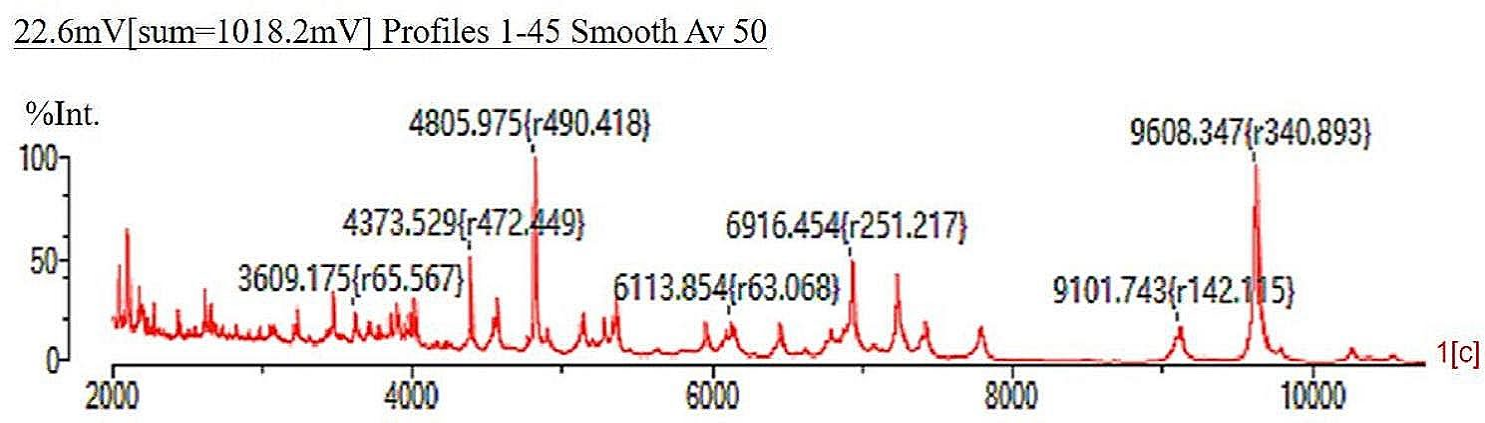
The identification mass spectrum of the test bacteria *Note*: The X-axis represents the mass-to-charge ratio (M/z), which is the ratio of the mass of the ion to its charge, and the Y-axis represents the intensity of the ion signals detected by the detector. It exhibits characteristic peak spectra at around 2100 MHz, 4400 MHz, 4800 MHz, 6900 MHz, and 9600 MHz.

Mass spectrometry analysis was performed using Matrix-Assisted Laser Desorption/Ionization Time-of-Flight (MALDI-TOF) technology to obtain the mass spectrum. Further identification was conducted using the VITEK MS fully automated rapid microbial mass spectrometry detection system. The bacteria was identified as *Vagococcus fluvialis* (with a confidence level of 99.9%). It exhibits characteristic peak spectra at around 2100 MHz, 4400 MHz, 4800 MHz, 6900 MHz, and 9600 MHz. As shown in Fig. 3.

### Drug sensitivity test results

The VITEK-2Compact fully automated microbial susceptibility analysis system indicated that *Vagococcus fluvialis* is most sensitive to tigecycline, vancomycin, ciprofloxacin/levofloxacin, linezolid, and exhibits intermediate sensitivity to erythromycin, levofloxacin, ciprofloxacin, ampicillin/sulbactam, tetracycline, among other antibiotics. There is a synergistic effect observed when high-level streptomycin is combined with vancomycin, and it shows sensitivity. However, it has poor inhibition effect against penicillin and furantoin.(Table 1).

**Table 1:** Antimicrobial susceptibility of the *Vagococcus flfluvialis* strain isolated from patient

	Antimicrobial	MIC (µg/ml)
1	Penicillin	≥ 64
2	Amoxicillin	16.0
3	High-level synergy of gentamicin	SYN-R
4	High-level synergy of lincomycin	SYN-S
5	Ciprofloxacin	8.0
6	Levofloxacin	8.0
7	Erythromycin	8.0
8	Quinupristin/Dalfopristin	1.0
9	Linezolid	1.0
10	Vancomycin	≤ 0.5
11	Tetracycline	16.0
12	Tigecycline	≤ 0.12
13	Furazolidone	64.0
14	Ceftriaxone	≤ 0.5

Note: MIC means Minimum Inhibitory Concentration; S means susceptible, R means resistance; SYN-S means high-level synergistic test susceptible; SYN-R means high-level synergistic test resistance

## Discussion

To date, there have been no reports of culturing *Vagococcus fluvialis* from human urine specimens, and there are very few reports of human infections caused by *Vagococcus fluvialis*: In 1997, Teixeira [3] reported the isolation of 4 strains of riverine *Vagococcus fluvialis* from human clinical specimens, most of which were initially classified as unidentified enterococci. Teixeira confirmed them as *Vagococcus fluvialis* through the study of phenotypic and genotypic characteristics of the isolates, providing the molecular characteristics of *Vagococcus fluvialis* and the first evidence of their possible association with human infections. In 2008 [4], Ali Al-Ahmad isolated *Vagococcus fluvialis* from filled teeth with periapical lesions. In 2019 [9], Jadhav reported a case of *Vagococcus fluvialis* infective endocarditis. In 2019 [11], Zhou reported the isolation of *Vagococcus fluvialis* from postoperative infection puncture fluid in the lower segment of the left femur in humans. In 2020 [12], Kucuk reported a case of *Vagococcus fluvialis* isolated from ascites in a patient with liver cirrhosis. In 2023 [15], Wang first reported the isolation and identification of *Vagococcus fluvialis* from gallbladder puncture fluid in patients with chronic cholecystitis. *Vagococcus fluvialis* can also be isolated from various injuries in pigs, cows, cats, tonsils, and the tonsils of horses [20]. It is a Gram-stain positive, catalase-negative cocci. Most of the strains isolated from injury cases were obtained from animals with conditions unrelated to *Vagococcus* infections. Only a portion of *Vagococcus fluvialis* strains are motile. Many strains produce positive reactions in the V-P test, alkaline phosphatase, and leucine arylamidase test, or ferment lactose and D-tagatose to produce acid. It has been reported that this bacterium causes severe damage to rainbow trout in fish farming at low water temperatures [21]. Given that *Vagococcus fluvialis* can cause infections in both humans and animals, and can be isolated from clinical specimens, it is often misidentified or overlooked in clinical laboratories due to diagnostic challenges. Therefore, when isolating suspected *Vagococcus fluvialis-like* bacteria from various specimens, it is important to differentiate them from relevant bacterial species and perform accurate identification. Meanwhile, we also discovered some advanced materials for precise detection of bacteria, as well as the latest nanomedical approaches for treating bacterial infections, which have propelled new strategies for accurate diagnosis and treatment.

We searched for all cases of human infection with *Vagococcus* in the PubMed database, Embase database, Google Scholar, China National Knowledge Infrastructure (CNKI), and Wanfang Data. Table 2 shows cases of the previously reported *Vagococcus* infections in the Literature in humans.

**Table 2:** Cases of the previously reported *Vagococcus* infections in the Literature in humans

Ref.	Year of Publication	Age (Years)/Sex	Isolated specimen	Previous diseases/Risk Factors	Duration of Symptoms Before Diagnosis	Diagnosis	Treatmen	Prognosis
Teixeira [3]	1997	N/A	Blood, peritoneal fluid, and wounds	N/A	N/A	Peritonitis, Skin and soft tissue infection	N/A	N/A
Ali Al-Ahmad [4]	2008	N/A	Root canal	N/A	N/A	Periradicular lesions	N/A	N/A
Schinmeister [5]	2009	N/A	Root canal	N/A	N/A	Periradicular lesions	N/A	N/A
Garci [6]	2016	58	Skin wound	Depressive disorder	N/A	Skin and soft tissue infection	Amoxicillin for 15 days	Complete resolution
Abuzaanona [7]	2016	34	Blood	Intravenous drug abuse	N/A	Infective endocarditis and embolic stroke	Ampicillin and Ceftriaxone for 6 weeks	Complete resolution
Raja [8]	2018	N/A	Blood	N/A	N/A	Infective endocarditis	N/A	N/A
Jadhav [9]	2019	70	Blood from patients with infective endocarditis	Coronary artery bypass graft、Aortic regurgitation	3 days	Infective endocarditis	Vancomycin was treated for 6 weeks after aortic valve replacement	Complete resolution
Shewmaker [10]	2019	N/A	Foot wound	N/A	N/A	Foot infection	N/A	N/A
Zhou [11]	2019	19	Infected femoral wound	Left femoral neck fracture、Fracture surgery	10 months	Postoperative wound infection	Expansion surgery and vancomycin bone cement filling	Partial resolution
Kucuk [12]	2020	55	Ascitic fluid	Liver cirrhosis	N/A	Spontaneous bacterial peritonitis	Antibiotic therapy	N/A
Matsuo [13]	2020	74	Blood from patients with decubitus ulcer infection	Decubitus ulcer	4 days	Bacteremia	Piperacillin/tazobactam and vancomycin for 4 weeks	Partial resolution
Altintas [14]	2020	78	Blood	Recurrent urinary tract infections, hysterectomy, hypertension and a cataract operation	4 days	Skin and soft tissue infection	piperacillin/tazobactam for 12 days	Complete resolution
Wang [15]	2023	66	Gallbladder puncture solution	Gastric lymphoma、Post-gastrectomy	N/A	Gallstones with chronic cholecystitis	Cholecystectomy and ceftazidime for a week	Complete resolution

Although *Vagococcus fluvialis* infections are currently extremely rare, these research reports indicate that if timely and appropriate antimicrobial treatment is not given in clinical practice, it can lead to bloodstream infections, and in severe cases, it can be life-threatening. Studies have shown that *Vagococcus fluvialis* express a large number of proteins related to known Gram-stain positive bacterial pathogenic and virulence factors, among which enolase and phosphoglycerate kinase are highly expressed, providing further directions for the study of the pathogenic mechanisms of *Vagococcus fluvialis* [1].

Many literature reports indicate that the main pathogens causing urinary tract infections are *Escherichia coli*, followed by *Staphylococcus spp*., *Pseudomonas aeruginosa*, *Klebsiella spp*., and *Proteus spp*. However, cases of *Vagococcus fluvialis* causing infections are extremely rare. In this study, preliminary verification of the pathogenic bacteria belonging to the rare genus of *Vagococcus fluvialis* was conducted through colony morphology observations after blood agar plate cultivation, Gram-stain microscopic analysis, automated microbiological detection instrument bioassays. To ensure accurate identification of the pathogenic bacteria, in combination with relevant literature reports, matrix-assisted laser desorption/ionization time-of-flight mass spectrometry (MALDI-TOF MS) was used, which has significant advantages in identifying rare bacterial species [10, 22]. In this study, MALDI-TOF MS technology was also utilized for identification and the results once again confirmed it to be *Vagococcus fluvialis* (with a confidence level of 99.9%).

So far, there is no standard method or criteria for drug sensitivity testing of *Vagococcus fluvialis*. In this study, the drug sensitivity test of enterococci was used as a reference, which suggests the need for more clinical research to standardize drug sensitivity testing for this bacterium. The patient in this case received cephalosporin treatment for infection upon admission, and based on the patient’s condition and the results of various infection indicators such as blood routine, C-reactive protein, cytokines, and procalcitonin, it was considered that the patient’s infection was still manageable. Postoperative re-examination of urine culture showed no bacterial or fungal growth, and other infection indicators were within normal range. The patient improved and was discharged one week after surgery.

One of the principles of using antimicrobial drugs is that they should not be changed or discontinued arbitrarily. Generally, antimicrobial drugs need to be taken for 2–3 days to reach a certain drug concentration in the body before they take effect. Changing drugs at will is not conducive to the effectiveness of the drugs. The use of antimicrobial drugs should pay attention to the correct drug level. However, not all diseases require high-level drugs. As long as the selected drugs are appropriate, even low-level drugs can exert sufficient effects. Excessive use of high-level drugs can lead to bacterial resistance and affect the efficacy of treatment for severe infections. The selection of appropriate antimicrobial drugs should be based on the characteristics and severity of the infection, and the effects and effectiveness of antimicrobial drugs on pathogenic bacteria are closely related to the characteristics and severity of the disease [23, 24]. Therefore, this case has certain clinical value for diagnose and the rational use of antimicrobial drugs in patients with *Vagococcus fluvialis* infections.

## Data Availability

No datasets were generated or analysed during the current study.
